# Improving the safety of the Manchester triage system for children with congenital heart disease

**DOI:** 10.1007/s00431-022-04594-6

**Published:** 2022-08-27

**Authors:** Franziska Leeb, Ursula Sharma, Lusine Yeghiazaryan, Henriëtte A. Moll, Susanne Greber-Platzer

**Affiliations:** 1 Division of Pediatric Pulmonology, Allergology and Endocrinology, Department of Pediatrics and Adolescent Medicine, Medical University Vienna, Waehringer Guertel 18-20, 1090 Vienna, Austria; 2 Center for Medical Statistics, Informatics and Intelligent Systems, Institute of Medical Statistics, Medical University Vienna, Spitalgasse 23, 1090 Vienna, Austria; 3 Department of General Paediatrics, Erasmus MC-Sophia Children’s Hospital, Wytemaweg 80, 3015 Rotterdam, CN Netherlands

**Keywords:** Emergency medicine, Manchester triage system, Safety, Modification, Children with congenital heart disease

## Abstract

This study is a prospective evaluation of the validity of a Manchester triage system (MTS) modification for detecting under-triaged pediatric patients with congenital heart disease (CHD). Children with CHD visiting the emergency unit of the Department of Pediatrics and Adolescent Medicine, University Hospital Vienna in 2014 were included. The MTS modification updated the prioritization of patients with complex syndromic diseases, specific symptoms related to chronic diseases, decreased general condition (DGC), profound language impairment, unknown medical history, or special needs. A four-level outcome severity index based on diagnostic and therapeutic interventions, admission to hospital, and follow-up strategies was defined as a reference standard for the correct clinical classification of the MTS urgency level. Of the 19,264 included children, 940 had CHD. Of this group, 266 fulfilled the inclusion criteria for the modified triage method. The MTS modification was significantly more often applied in under-triaged (65.9%) than correctly or over-triaged (25%) children with CHD (*p*-value *χ*^2^ test < 0.0001, OR 5.848, 95% CI: 3.636–9.6).

*Conclusion:* The MTS urgency level upgrade modification could reduce under-triage in children with CHD. Applying a safety strategy concept to the MTS could mitigate under-triage in such a high-risk patient group.

**Table:** 

**What is Known:** *• The Manchester triage system is considered to be valid and reliable but tends to over-triage.* *• A study by Seiger et al. showed poor performance in children with chronic illnesses, especially in children with cardiovascular diseases.*	
**What is New:** *• The MTS modification with one urgency level upgrade could decrease under-triage in children with congenital heart disease.* *• As reference standard a four level outcome severity index (OSI) was established to include diagnostic investigations, medical interventions, hospital admission or follow up visits in the assessment.*

**What is Known:**

*• The Manchester triage system is considered to be valid and reliable but tends to over-triage.*

*• A study by Seiger et al. showed poor performance in children with chronic illnesses, especially in children with cardiovascular diseases.*

**What is New:**

*• The MTS modification with one urgency level upgrade could decrease under-triage in children with congenital heart disease.*

*• As reference standard a four level outcome severity index (OSI) was established to include diagnostic investigations, medical interventions, hospital admission or follow up visits in the assessment.*

## Introduction

Triage systems are essential tools emergency units implement when at full capacity to safely and promptly manage patient flow according to clinical priority. The Manchester triage system (MTS) is a five-level triage algorithm based on general and symptom-based flowcharts and discriminators to determine the urgency level (UL) [1]. Each UL is associated with a maximum waiting time: immediate (0 min), very urgent (up to 10 min), urgent (up to 60 min), standard (up to 120 min), and not urgent (up to 240 min) [2, 3]. In previous studies, reference standards with three or five classes based on vital signs, diagnosis, diagnostic and therapeutic interventions, life-threatening conditions, admission to hospital, and follow-up data were used to evaluate the MTS in pediatric patients [4–6]. The validity of the MTS in pediatric emergency care is deemed moderate with a tendency to over-triage compared. Its sensitivity and specificity to identify high-urgency patients were 0.63 and 0.79, respectively [4].

Further studies evaluated modifications of the MTS in pediatric patients [7–9]. Thus, the discriminator “petechiae” was included to the category “very urgent” in the flowchart “rashes” to classify pediatric patients suspected to have a meningococcal infection [7]. In addition, the general discriminator “fever” used as “hot” (*T* > 38 °C) was downgraded from MTS UL 2 to MTS UL 3 for children aged > 3 months. In the study from van Veen et al., age-related modifications showed low performance in the improvement of the MTS assessment in children, with a slight increase for the specificity (0.79 vs. 0.87), but with virtually no change in the sensitivity (0.63 vs. 0.64) [7]. Also, the implementation of vital signs (heart rate, respiratory rate, and/or capillary refill time) in specific flowcharts showed only minimal improvement in the MTS performance [8]. Further adaptations for specific discriminators with possible misclassification were performed; for example, the discriminator “wheeze” was upgraded from MTS UL 4 to MTS UL 3, whereas the discriminator “unable to talk in sentences” was downgraded from MTS 2 to MTS 3. As a result, these modifications improved the classification regarding admissions to hospital with an increase for very urgent patients and consistent rates for those with low-risk ULs [9].

To date, studies on the MTS for chronically ill children have registered poor performances and a higher under-triage risk in this category [10, 11]. An early warning scoring tool for inpatient children, known as the Cardiac Children’s Hospital Early Warning Score (C-CHEWS), can detect deterioration and prevent cardiopulmonary arrest [12].

Congenital heart defects account for nearly one-third of all severe congenital anomalies in Europe, with prevalence estimates of 8 per 1000 live births [13, 14].

Pediatric patients with CHD seem to be at high risk for cardiopulmonary insufficiency and rapid deterioration, especially those with infections or additional underlying conditions. A highly sensitive and specific scoring tool is therefore crucial for this patient group [11, 12].

Currently, there is no validated emergency unit scoring system for children with CHD or most other chronic diseases [11], and high-risk chronic diseases are underrepresented in the MTS. Only four specific discriminators describe a chronic disease (cardiac, allergic, respiratory, hematological history) and one indicates immunosuppression in few flowcharts (chest pain and palpitations, allergy, collapsed adult, rashes, shortness of breath in children, unwell baby, unwell child, urinary problems, and concerned parents) [1–3]. Thus, it seems necessary for the MTS to integrate first assessment guidelines for chronically ill patients [11].

Congenital heart defects represent a main focus (1/3 of all chronic diseases) at the emergency unit of the Department of Pediatrics and Adolescent Medicine at the Medical University, Vienna. Therefore, a safe and swift approach should be guaranteed.

This validation study aimed to investigate over-triage, correct triage, and under-triage in CHD patients, the application of well-defined urgency level upgrade criteria, and compare the reference system to the original MTS levels.

## Methods

### Study design and study population

This validation study gathered 23,258 patients who visited the emergency unit of the Department of Pediatrics and Adolescent Medicine, at the University Hospital Vienna, from January to December 2014. Self-referred patients up to 18 years are primarily treated, and approximately 25% of them present with chronic illnesses including congenital heart defects, inborn errors of metabolism, nephrology conditions, gastrointestinal, hepatic, or endocrine disorders, pulmonary, neurological, psychosomatic, or autoimmune diseases, and brain tumors, underwent organ transplantation, or are preterm infants.

Exclusion criteria were age ≥ 18 years (*n* = 74), or no record of the patient’s MTS urgency level (*n* = 1189), with missing diagnosis (*n* = 2722), or no information on the follow-up procedure (*n* = 9). Additionally, the MTS UL modification was introduced for patients with specific symptoms related to a chronic disease or other special features. The included children (*n* = 19,264) were divided into two groups, one without (*n* = 15,843) and the other with chronic diseases (*n* = 3421). The group with congenital heart disease (CHD) consisted of 940 children (27.5%). Children with a CHD were categorized according to the 10th version of the International Classification of Diseases Code (ICD-10) using the EUROCAT (European Registration of Congenital Abnormalities and Twins) as the reference for CHD [15]. The children with CHD were sorted as follows: cyanotic heart defects (corrected/uncorrected/palliative), acyanotic heart defects (corrected/uncorrected/partially corrected), acquired heart defects, inflammatory heart diseases, cardiac insufficiency, cardiomyopathy, arrhythmia, or heart transplantation.

### MTS and modification

Since 2012, the emergency unit of the Department of Pediatrics and Adolescent Medicine, Medical University Vienna has been using the 3rd German version of the international Manchester triage book published as Emergency Triage by the Manchester Triage Group (3rd edition) [1, 2]. The MTS one-urgency level upgrade modification was introduced in 2013. The MTS was applied by a pool of 17 triage nurses with a minimum of 3 years of extensive experience in pediatric emergency and an MTS basic course certification. In 2013 and 2014, the annual MTS audits showed 77.9% and 84.0% accuracy, and 11.2% and 5.8% incompleteness in random samples of 160 and 168 triage documentations, respectively.

The MTS modification with one-UL upgrade was used to assess chronically ill children presenting with cyanosis (oxygen saturation) and heart defects, abdominal extension or vomiting in gastrointestinal, metabolic, or neurological diseases, as well as in preterms, and patients with complexity related to multiple abnormalities. In addition, the presence of one of the following factors automatically qualified the patients for the one-UL upgrade: speech disorders, unknown clinical history, decreased general condition (DGC), or special needs (psychiatric disorders or neurodevelopmental delay).

### Data analysis

A four-level outcome severity index (OSI), similar to existing reference standard classification systems [3–5, 16], was developed to evaluate the validity of the MTS at the emergency unit of the Department of Pediatrics and Adolescent Medicine, University Hospital Vienna. The OSI ranked priority based on diagnostic investigations (laboratory tests, chest radiography, ultrasonography, electrocardiogram, echocardiography, computed tomography scan), medical interventions at the emergency unit (e.g., intravenous medication or fluid, inhalation, nebulization, monitoring), hospital admission (intensive care unit (ICU) or inpatient ward), or a follow-up visit at the outpatient clinic or pediatrician’s office. Children needing diagnostic investigations, hospital admission, or life-saving interventions were assigned a higher OSI level and presented with a more severe illness.

The highest level, OSI 1, was assigned if ICU admission or life-saving interventions and hospitalization were required. OSI 2 was indicated in case of hospital admission or interventions and follow-up at the outpatient clinic. OSI 3 was assigned to patients needing diagnostic investigations or medical interventions with follow-up at the outpatient clinic or the pediatrician’s office. The lowest level, OSI 4, implied that no treatment at the emergency unit was required and follow-up at the pediatrician’s office was sufficient.

Correct triage was indicated, if the MTS urgency level (MTS UL) corresponded to the assigned outcome severity index (OSI); this means MTS UL 1 = OSI 1, MTS UL 2 = OSI 1 or OSI 2, MTS UL 3 = OSI 2 or OSI 3, MTS UL 4 = OSI 3 or OSI 4, and MTS UL 5 = OSI 4. Matching the MTS urgency levels (ULs) to the outcome classification optimized initial assessment accuracy. Under-triage occurred when the MTS UL was lower than the assigned OSI, whereas over-triage occurred when the MTS UL was higher than the assigned OSI.

### Statistical analysis

Absolute and relative frequencies were calculated for each MTS UL and OSI classifications as well as for the under-triaged, over-triaged, and correctly triaged patients.

To capture the under-triage decrease rate in CHD patients, we constructed a frequency table and performed a *χ*^2^ test.

We calculated the absolute and relative frequencies of original MTS patients, patients with MTS one-level upgrade modification, and all CHD patients to analyze the CHD subgroups.

The *χ*^2^ test was performed to evaluate differences between MTS original and MTS modification subjects with regard to the subgroups.

### Data collection

Patients’ data were recorded by either manual-data entry or electronically imported. Data from additional examinations were registered in the electronic patient information system. Only authorized staff had access to the data collection. The Ethics Committee of the Medical University of Vienna, Austria approved this study (no. 1405/2014). Data on the original MTS and clinical procedures were part of a large international study called TrIAGE, conducted by Henriëtte A. Moll, Department of General Pediatrics, Erasmus MC—Sophia Children’s Hospital, Rotterdam, CN, Netherlands. The TrIAGE study included no data on the MTS modification, the outcome severity index (OSI), the types of congenital heart diseases (Medical Ethics Committee Erasmus MC (MEC-2013–567)), the Maasstad Ziekenhuis Board of Directors (Protocol L2013-103), the Joint Research Compliance Office (JRCO), Imperial College London (reference number: 14SM2164, Ethics Committee Reference Number 14/WA/1051), or the Comissão de Ética para a Saúde do Hospital, Prof. Dr. Fernando Fonseca, EPE (Reunião de 06 de Dezembro de 2017).

## Results

In total, 3412 children and adolescents with chronic diseases were triaged using the MTS in the pediatric emergency unit from January to December 2014. Of these, 940 (100%) had a congenital heart disease (CHD). Among the CHD patients, 458 (48,7%) were females. The five age groups comprised ten newborns (up to 28 days), 221 infants (> 28 days to < 1 year), 351 toddlers (≥ 1 year to < 4 years), 224 school-aged children (≥ 4 years to < 10 years), and 134 adolescents (≥ 10 years to < 18 years). With the MTS modification, 674 patients with CHD remained in the original MTS UL group, and 266 met clinically relevant criteria for the one-UL upgrade. Hospitalization was indicated for 167 (17.7%) CHD cases, six of whom had to be admitted to the intensive care unit.

Under-triaged patients were 82 (8.7%), and 54 (2/3) were upgraded; whereas correctly or over-triaged ones were 858 (91.3%), 212 (1/4) were upgraded.

**Fig. 1:**
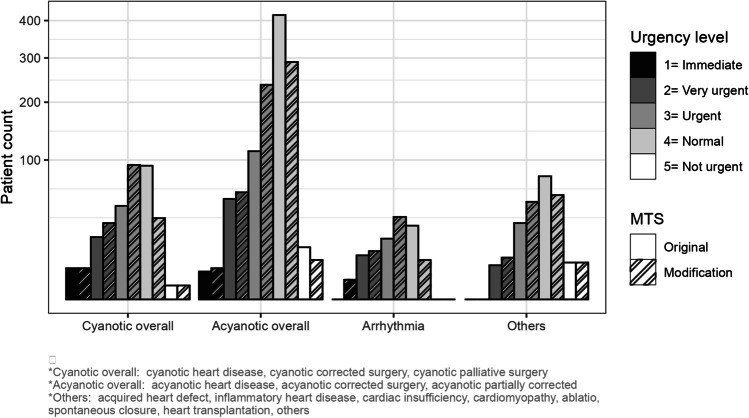
Urgency levels of MTS original and MTS modified as final classification related to CHD subgroups (*n* = 940)

According to the original MTS, nine CHD patients were assigned the highest MTS UL (UL 1, immediate). Of these, five had a cyanotic heart disease, three had an uncorrected acyanotic heart disease, and one had an acyanotic partially corrected heart disease. For five patients, admission to the intensive care unit was necessary and four were admitted to the inpatient ward. The MTS modification correctly up-triaged two patients with arrhythmia and one with acyanotic heart disease to MTS 1. Further details about the subgroups and MTS classification 1–5 (original/modification) are described in Fig. 1 (MTS original and final categorization).

Among the 266 (100%) CHD subjects requiring upgrade, three (1.1%) were redistributed to the UL 1 (immediate), 25 (9.4%) to UL 2 (very urgent), 232 (87.2%) to UL 3 (urgent), and six (2.3%) to UL 4 (standard). Table 1 reports the demographic data of the cumulative study population and congenital heart disease patients.

**Table 1:** Demographic data of the total study population and children with congenital heart disease

Demographics	*n* (%)
**Total study population** **No chronic disease** **Chronic diseases** Congenital heart disease Other chronic diseases	19,264 (100) 15,843 (82.2) 3421 (17.8) 940 2481
**Congenital heart disease** **Sex** Female **Age distribution** Newborn (≤ 28 days) Infant (> 28 days to < 1 year) Toddler (≥ 1 year to < 4 years) Schoolchild (≥ 4 years to < 10 years) Adolescent (≥ 10 years to < 18 years) **Diagnostic examination** Yes **Intervention** Yes **Life-saving intervention** Yes **Follow-up** ICU admission Hospital admission Outpatient clinic Pediatrician	940 (100) 458 (48.7) 10 (1.1) 221 (23.5) 351 (37.3) 224 (23.8) 134 (14.3) 463 (49.3) 228 (24.3) 4 (0.4) 6 (0.6) 161 (17.1) 99 (10.5) 674 (71.7)

The three (1.1%) patients upgraded from UL 2 to UL 1 (immediate) needed additional diagnostic tests. One patient needed acute intervention and admission to the intensive care unit, another was admitted to the inpatient ward, and the third could be discharged after self-limitation of a tachyarrhythmia, in stable condition and with an immediate follow-up appointment at the outpatient pediatric cardiac clinic.

Of the 25 (9.4%) patients upgraded from UL 3 (urgent) to UL 2 (very urgent), 22 (8.2%) needed diagnostic tests, and 12 (4.5%) interventions. Hospital admission was necessary for 14 patients (5.2%), while 11 (4.1%) could be discharged and scheduled for a follow-up visit at the pediatrician’s office (nine patients) or in the outpatient clinic (two patients).

The largest group, 232 patients (87.2%), was upgraded from UL 4 (standard) to UL 3 (urgent). Diagnostic tests were performed on 134 patients (50.4%), 59 needed interventions (22.2%), and 49 admission to the hospital (18.4%). One hundred eighty-three patients (68.8%) could be discharged; of these, 35 (13.2%) were considered for a follow-up visit in the outpatient clinic, and 148 (55.6%) at the pediatrician’s office.

Out of 22 patients classified as UL 5 (not urgent), six (2.3%) were reconsidered as UL 4 (standard). Only one needed an additional diagnostic examination. All six patients could be discharged and referred to the pediatrician’s office for a follow-up visit (Table 2).

**Table 2:**
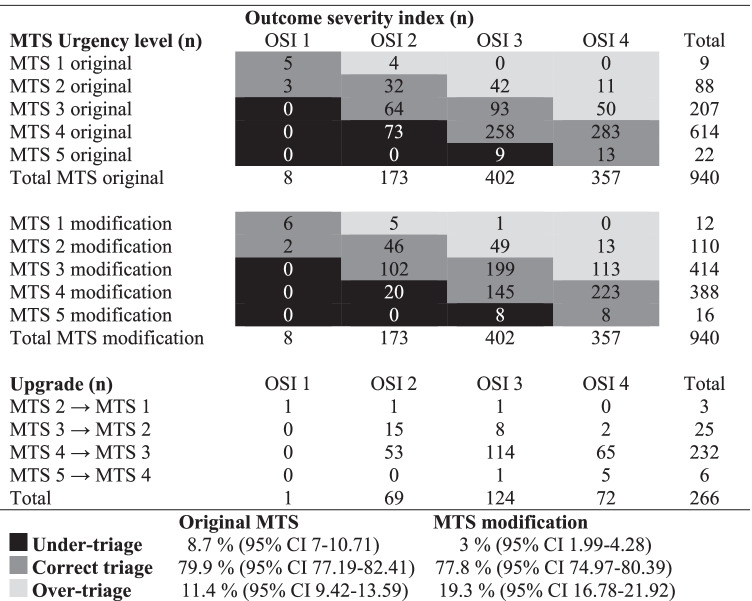
Distribution of the Manchester triage system (MTS) urgency level and outcome severity index (OSI) for children with congenital heart disease. Stated are the numbers of the original MTS and the MTS modification, the number of one-urgency level upgrading, and the calculated value and percent of correct, over-triage, and under-triage

The MTS one-UL modification in children with CHD decreased under-triage by two-thirds, from 82 patients (8.7%, only original MTS) to 28 (3%; remaining after MTS modification), and increased over-triage, from 107 patients (11.4%; only original MTS) to 181 (19.3%; increase after MTS modification).

The MTS modification proved to be most effective for children assigned to OSI 2 (*n* = 173) as under-triage rates plummeted from 73 patients (42.2%) to 20 (11.6%), correctly triaged went from 96 (55.5%) to 148 (85.6%), whereas over-triage remained almost constant (four; 2.3% with original MTS vs. five; 2.9% with modified MTS). Conversely, the MTS modification doubled the over-triage cases of patients assigned to OSI 4 (*n* = 357), from 61 (17.1%) with the original MTS to 126 (35.3%) with the modified MTS (Table 2).

It could be shown that pediatric patients with CHD often need hospital admission or interventions and follow-up at the outpatient clinic, procedures in agreement with OSI 2. This means that children with CHD are on a higher risk for clinical deterioration, and if presenting to the ED there is a higher need for clinical resources.

Under-triaged patients with CHD were more likely to be upgraded with the MTS modification than correctly or over-triaged ones [OR 5.767 (95% CI: 3.585–9.465); *p*-value *χ*^2^ test < 0.0001]. Table 3 shows the detailed data of patients with congenital heart disease defined as under-triaged and correctly/over-triaged with reference to the original Manchester triage system (MTS) and the MTS one-UL modification.

**Table 3:** Cross table for children with congenital heart disease defined as under-triage and correct/over-triage related to the original Manchester triage system (MTS) and the MTS modification with one-level upgrading

Children with congenital heart disease	Original MTS *n* (%)	MTS modification with one-level upgrade *n* (%)	Sum *n* (%)
Under-triage (original MTS) Correct-triage (original MTS) Over-triage (original MTS) Sum	28 (3.0) 542 (57.6) 104 (11,1) 674 (71.7)	54 (5.7) 209 (22.3) 3 (0,3) 266 (28.3)	82 (8.7) 751 (79.9) 107 (11.4) 940 (100)

OR 5.848 (95% CI: 3.636–9.6); *p*-value *X*^2^ test < 0.0001

Table 4 includes a more in-depth analysis of the age distribution and CHD subgroups. Infants were more often upgraded by one UL (20.9% (original MTS) to 30.1% (modified MTS)), while adolescents tended to be assessed more often with the original MTS (16.8% (original MTS) to 7.9% (modified MTS)). CHD subgroups comprised cyanotic heart diseases (corrected/uncorrected/palliative), acyanotic heart diseases (corrected/uncorrected/partially corrected), acquired heart defects, inflammatory heart defects, cardiac insufficiency, cardiomyopathy, arrhythmia, or heart transplantation.

**Table 4:** Cross table with age and subgroup for children with congenital heart disease compared the original Manchester triage system (MTS) to the MTS modification with one-level upgrading

Children with congenital heart disease	Original MTS *n* = 674 (%)	MTS modification with one-level upgrade *n* = 266 (%)	Total *n* = 940 (%)
**Age distribution (mean)** **Newborn** (≤ 28 days old) **Infant** (> 28 days old to < 1 y.o.) **Toddler** (≥ 1 y.o. to < 4 y.o.) **School kid** (≥ 4 y.o. to < 10 y.o.) **Adolescent** (≥ 10 y.o. to < 18 y.o.) **CHD subgroup** Cyanotic heart disease Cyanotic corrected surgery Cyanotic palliative surgery Acyanotic heart disease Acyanotic corrected surgery Acyanotic partially corrected Spontaneous closure Arrhythmia Cardiomyopathy Acquired heart disease Inflammatory heart disease Heart transplantation Cardiac insufficiency Ablation Others	4.26 (SD ± 4.8) 8 (1.2) 141 (20.9) 251 (37.2) 161 (23.9) 113 (16.8) 8 (1.2) 61 (9.0) 26 (3.9) 310 (46.0) 99 (14.7) 43 (6.4) 20 (3.0) 31 (4.6) 12 (1.8) 23 (3.4) 1 (0.1) 9 (1.3) 1 (0.1) 5 (0.7) 25 (3.7)	2.98 (SD ± 3.7) 2 (0.8) 80 (30.1) 100 (37.6) 63 (23.7) 21 (7.9) 8 (3.0) 32 (12.0) 28 (10.5) 123 (46.2) 16 (6.0) 8 (3.0) 2 (0.8) 26 (9.8) 3 (1.1) 3 (1.1) 0 (0) 3 (1.1) 1 (0.4) 0 (0) 13(4.9)	3.92 (SD ± 4.6) 10 (1.1) 221 (23.5) 351 (37.3) 224 (23.8) 134 (14.3) 16 (1.7) 93 (9.9) 54 (5.7) 433 (46.1) 115 (12.2) 51 (5.4) 22 (2.3) 57 (6.1) 15 (1.6) 26 (2.8) 1 (0.1) 12 (1.3) 2 (0.2) 5 (0.5) 38 (4.0)

*p*-value *X*^2^ test < 0.0001

As expected, children with cyanotic heart defects, even after palliative heart surgery, or with arrhythmia were significantly more often upgraded by one UL. In addition, however, 46% of children with arrhythmias classified for one-UL upgrade. These patients were significantly more often upgraded by one UL than those with acyanotic heart diseases with or without a corrective heart surgery or spontaneous closure, as shown in Table 4 (*p*-value *χ*^2^ test < 0.0001). Nevertheless, 28% of children with an acyanotic heart disease needed the one-UL upgrade.

## Discussion

The Manchester triage system is a valid and reliable triage tool in pediatric emergency units but tends to over-triage [4–6]. As the assessment of chronically ill children pivots on safety and reliability, the need for validated triage systems for this patient group is of utmost importance [10–12]. Seiger et al. proved that children with a chronic heart disease had a higher risk for under-triage (24.9%) than children without (11%) [11].

Adaptations of the triage system for certain vulnerable pediatric patients with regard to specific chronic diseases, clinically relevant criteria, or other special features seem to be a practical approach to prevent under-triage and increase correct triage, especially in children with CHD [8, 17]. Therefore, the pediatric emergency unit of the Department of Pediatrics and Adolescent Medicine, University Hospital Vienna adopted the MTS one-urgency level upgrade modification. This study could confirm that under-triage is a common problem in children with chronic diseases, especially congenital heart diseases [10, 11]. Our findings estimated the original MTS under-triage at 8.7% among children and adolescents with CHD, and revealed a reduction to 3% when implementing the MTS one-UL upgrade modification to assess patients with defined clinically relevant criteria or other special features (Table 3).

We can conclude that pediatric patients with CHD visiting the ED are at higher risk for clinical deterioration and therefore avoiding under-triage is essential. Under-triage means longer waiting time, for medical resources and clinical stabilization. MTS upgrading for symptoms of cyanosis or reduced general condition seems to be crucial in CHD.

The four-level outcome severity index (OSI) was developed as a reference standard for the correct triage, under-triage, and over-triage, and comprised additional clinical examinations and interventions, hospital admission, or outpatient follow-up. Under-triage was most frequent in patients who met the OSI 2 priority criteria and had to be re-triaged from the original MTS UL 4 to MTS UL 3. We observed that over-triage increased from 11.4 to 19.3% in the effort to prevent under-triage in children with CHD (Table 2). Ultimately, while avoiding under-triage remains a significant concern as a predictor of clinical deterioration, a slight increase in over-triage is inevitable. Therefore, patient safety and immediate initiation of treatment especially for children with cyanotic heart defects and arrhythmias are crucial to patient outcomes.

Zachariasse et al. proved that children aged < 3 months had the highest risk for under-triage (OR 2.87; 95% CI 2.00–4.10) [10]. The present study produced comparable evidence in newborns and infants with CHD but the modified MTS (20.9% from original MTS to 30.1% with MTS modification) was more often utilized in this group than in older children and adolescents. This strategy reduced or prevented under-triage in such a vulnerable cohort. Additionally, since chronically ill children appear to be underrepresented in the MTS flowcharts, chronic illnesses and clinically relevant criteria and features should be acknowledged in the triage assessment to improve patient safety and minimize under-triage. Limitations of this study may be the limited number (*n* = 266) of patients with CHD and MTS one-UL upgrade modification. A larger sample size would generate more accurate results and more precise statistical analysis of the CHD subgroups including cyanotic heart disease, acyanotic heart disease, arrhythmia, and other cardiac diseases.

## Conclusion

Children with CHD visiting the ED are more vulnerable for clinical deterioration and life-threatening events than previously healthy children. This fact should be taken into account at the MTS classification to improve clinical outcome in CHD.

The most evident effect to avoid under-triage was shown in patients with cyanotic heart disease or arrhythmias and in newborns and infants with CHD, who must be considered at particular health risk.

## Limitations and perspectives

A limitation of this study may be the limited number (*n* = 266) of patients with a wide spectrum of CHD with need for MTS one-UL upgrade modification.

For a larger sample size, multicenter studies can be required, but our results present the first data based on the MTS modification with one-level upgrading at our pediatric emergency unit and could show the reduction of under-triage in patients with CHD.

Ultimately, avoiding under-triage is the main focus for chronic ill patients and therefore it seems tolerable that a slight increase of over-triage occurs. For confirmation of this statement, further studies may be indicated.

## Data Availability

The study has associated data in a data repository of the Medical University of Vienna.

## Abbreviations

C-CHEWS: Cardiac Children’s Hospital Early Warning Score
CHD: Congenital heart disease
CI: Confidence interval
ICU: Intensive care unit
MTS: Manchester triage system
OR: Odds ratio
OSI: Outcome severity index
UL: Urgency level

## Code availability

Data available on request from the authors.
